# Supplementary material for: A Comparative Study on the Effects of the Lysine Reagent Pyridoxal 5-Phosphate and Some Thiol Reagents in Opening the Tl+-Induced Mitochondrial Permeability Transition Pore
Source: Int J Mol Sci. 2023 Jan 27;24(3):2460. doi: 10.3390/ijms24032460 (PMC9916919; doi:10.3390/ijms24032460)
Supplement: Supplementary file 1 [file ijms-24-02460-s001.zip › ijms-2156138-supplementary.pdf]

Supplementary Material

Supplementary data for the manuscript:

Comparative study effects of the lysine reagent pyridoxal 5-phosphate and some thiol reagents in opening the Tl^{+}-induced permeability transition pore in experiments in vitro with calcium-loaded rat liver mitochondria

Sergey M. Korotkov and Artemy V. Novozhilov

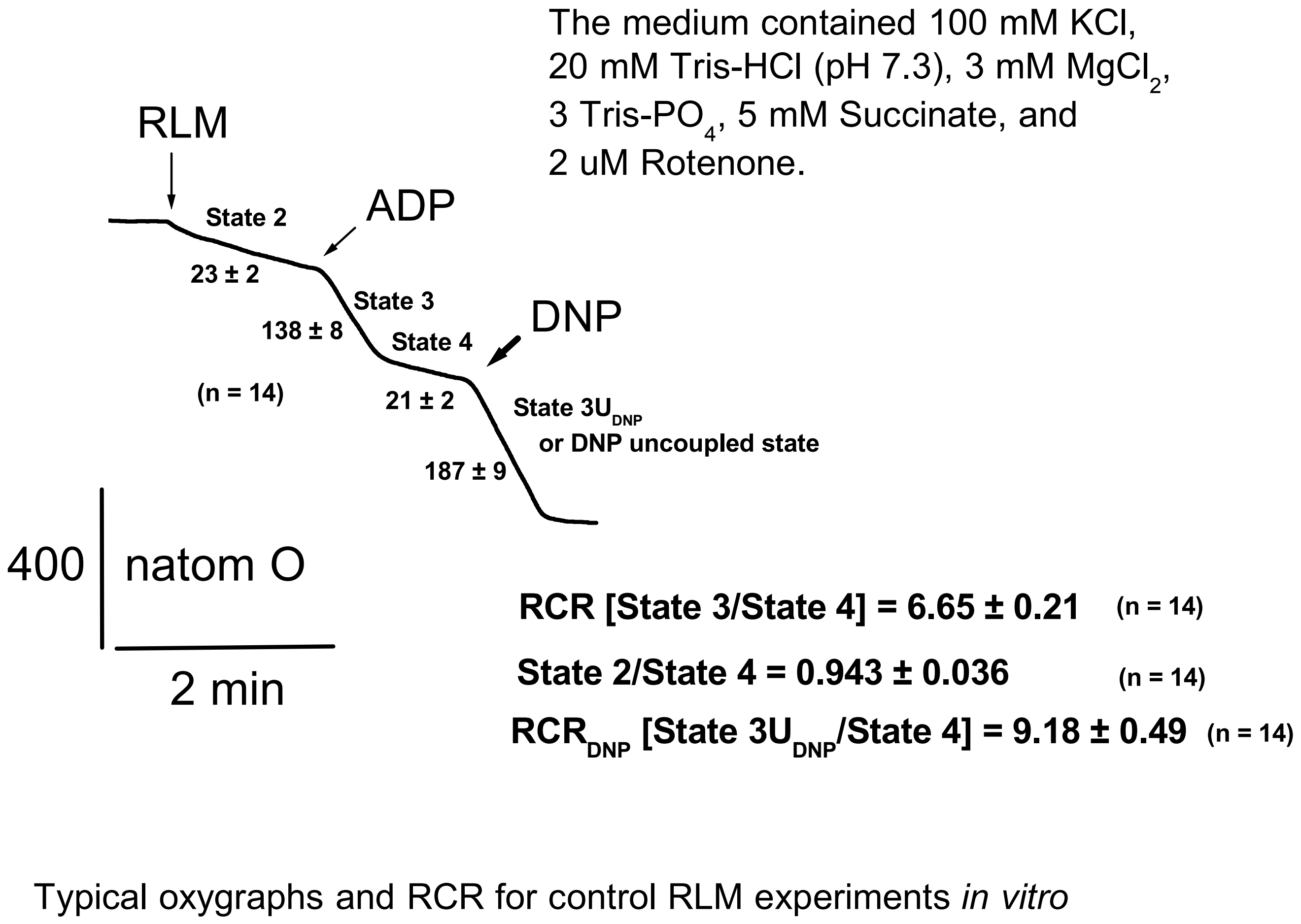

Figure S1. Typical oxygraph and RCR to control rat liver mitochondrial preparations.

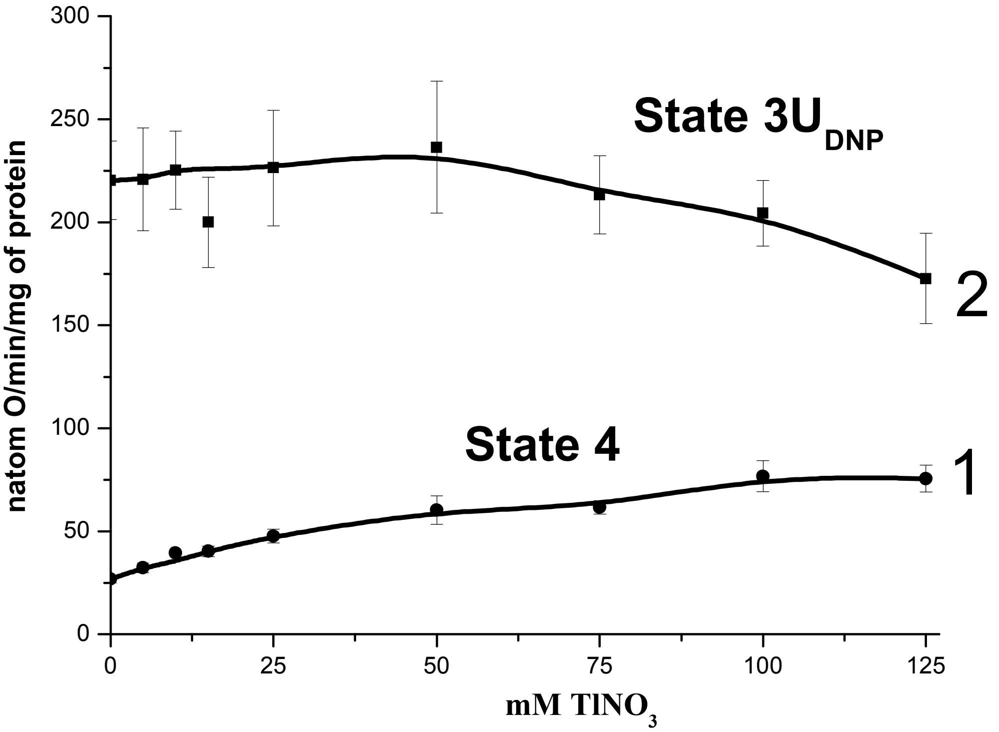

Figure S2. Effect of Tl^+ on oxygen consumption rates (natom O min/mg of protein) in succinate-energized rat liver mitochondria. Mitochondria (1.5 mg/mL of protein) were injected in sucrose-adjusted 280 mOsm medium containing 0-125 mM $TlNO_3$ (traces 1 and 2) and 5 mM Tris-NO_3 (pH 7.3), 5 mM succinate, 4 μM rotenone, 3 mM $Mg(NO_3)_2$, and 3 mM Tris-P_i . 2,4-dinitrophenol (DNP) of 30 μM was administered into the medium to trigger DNP-stimulated respiration (trace 2) after 2 min recording of state 4 (trace 1). Error bars were calculated by the Muller formula from rates found for three different mitochondrial preparations.

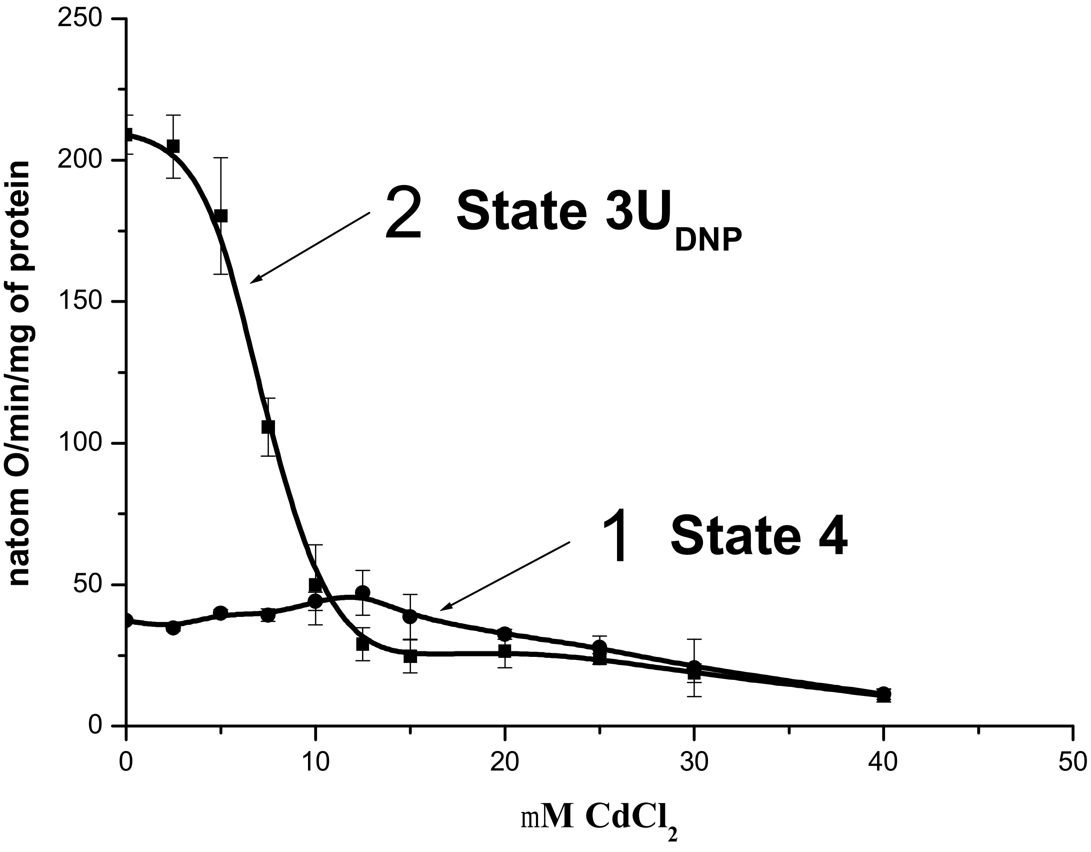

Figure S3. Effect of Cd^{2+} on oxygen consumption rates (natom O min/mg of protein) in succinate-energized rat liver mitochondria. Mitochondria (2 mg/mL of protein) were injected in 280 mOsm medium containing 100 mM KCl (traces 1 and 2), 20 mM Tris-HCl (pH 7.3), 5 mM succinate, 4 μM rotenone, 3 mM $MgCl_2$, and 3 mM Tris-P_i . 2,4-dinitrophenol (DNP) of 30 μM was administered into the medium to trigger DNP-stimulated respiration (trace 2) after 2 min recording of state 4 (trace 1). Error bars were calculated by the Muller formula from rates found for three different mitochondrial preparations.

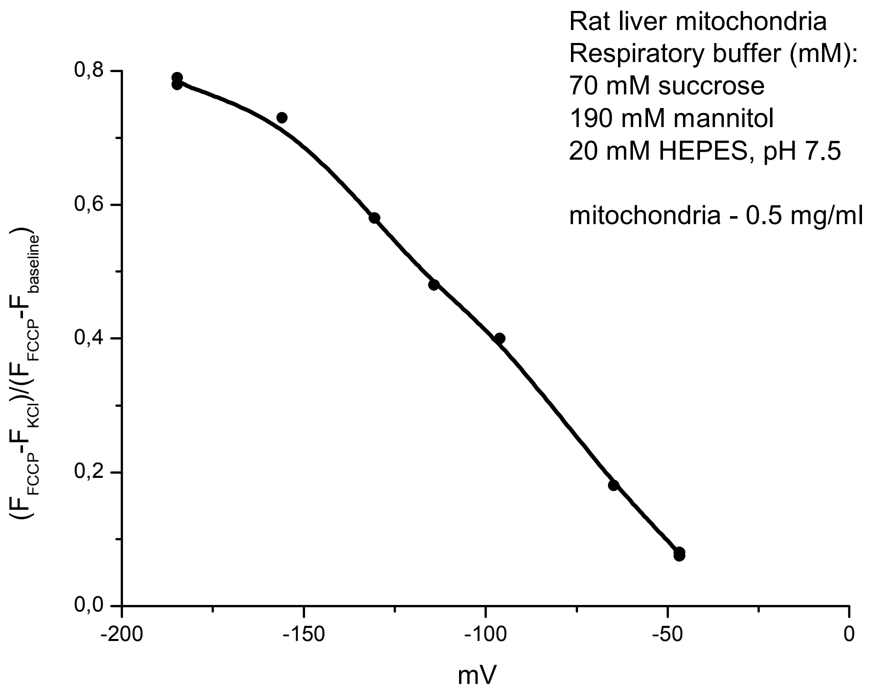

Figure S4. Calibration in the dependence of safranin fluorescence on ΔΨ. The safranin fluorescence was evaluated at 20 °C using a Shimadzu RF-1501 spectrofluorimeter (Shimadzu, Japan) at 485/590 nm wavelength (excitation/emission). To record $F_{baseline}$ (2 min), mitochondria (0.5 mg/ml of protein) were injected into a quartz transparent cuvette filled by 3 ml of the medium containing 190 mM mannitol, 70 mM sucrose, 5 μM rotenone, 2.5 μM safranin, 20 nM valinomycin, 4 μg/ml of oligomycin, and 20 mM HEPES, brought to pH 7.5 with 1 M NaOH. To register F_{KCl} (2 min), concentrated KCl solution was farther injected into the cuvette to reach final concentrations of 0.1, 0.3, 0.8, 1.5, 3, 10, and 20 mM K^+. Finally, 1 μM FCCP was added into the cuvette with subsequent 2 min registration of F_{FCCP}. From these data, $(F_{FCCP} - F_{KCl})/(F_{FCCP} - F_{baseline})$ was calculated for each measurement and plotted against ΔΨ, calculated using the Nernst equation.

Table S1ab

Effects of pyridoxal 5 phosphate (P5P) on swelling (ΔA_{540}) of succinate-energized rat liver mitochondria

**P5P** (mM)	**free of Ca^{2+}**		**100 μM Ca^{2+}**		**100 μM Ca^{2+} + ADP**	
	ΔA_{540} ± SEM	P value	ΔA_{540} ± SEM	P value	ΔA_{540} ± SEM	P value
0	-0.010±0.001 (9)	P < 0.01	-0.201±0.011 (9)	-	-0.009±0.001 (9)	P < 0.01
2	-0.008±0.001 (5)	P < 0.01	-0.199±0.019 (7)	*	-0.020±0.001 (6)	P < 0.01
4	-0.011±0.001 (5)	P < 0.01	-0.213±0.010 (7)	*	-0.022±0.004 (5)	P < 0.01
6	-0.014±0.001 (5)	P < 0.01	-0.233±0.010 (8)	P < 0.05	-0.053±0.010 (8)	P < 0.01

The changes in ΔA_{540} were detected within four minute interval after addition of mitochondria (see Figs. 1a and 1b) and presented as Means ± SEM. The number of experiments showed in parentheses. P-values were accordingly calculated to experiments free of P5P (A) or 100 μM Ca^{2+} (B). Asterisks indicate that statistical difference between appropriate ΔA_{540} values is not statistically significant.

Table S1c

Effects of 6 mM pyridoxal 5 phosphate (P5P) on swelling (ΔA_{540}) of succinate-energized and calcium-loaded rat liver mitochondria in the presence of MPTP inhibitors

**MPTP inhibitors**	ΔA_{540} ± SEM	P value
control (Ca^{2+} alone and free of additions)	-0.201±0.011 (9)	-
P5P	-0.233±0.010 (8)	P < 0.05
P5P + CsA	-0.248±0.010 (3)	P < 0.04
P5P + ADP	-0.053±0.010 (8)	P < 0.01
P5P + NEM	-0.046±0.009 (3)	P < 0.01
P5P + CsA + NEM	-0.045±0.008 (3)	P < 0.01
P5P + ADP + CsA	-0.014±0.006 (3)	P < 0.01
P5P + ADP + NEM	-0.018±0.004 (3)	P < 0.01

The changes in ΔA_{540} were detected within four minute interval after addition of mitochondria (see Figs. 1c) and presented as Means ± SEM. The number of experiments showed in parentheses. P-values were accordingly calculated to experiments with and 100 µM Ca^{2+} and free of P5P. Asterisks indicate that statistical difference between appropriate ΔA_{540} values is not statistically significant.

Table S1de

Effects of thiol reagents on swelling (ΔA_{540}) of succinate-energized rat liver mitochondria

thiol reagents	free of Ca^{2+}		100 µM Ca^{2+}	
	ΔA_{540} ± SEM	P value	ΔA_{540} ± SEM	P value
free of thiol reagents	-0.016±0.002 (9)	-	-0.205±0.007 (6)	-
6 mM P5P	-0.095±0.005 (8)	*	-0.261±0.010 (8)	P < 0.01
10 µM EMA	0.002±0.006 (3)	P < 0.05	-0.252±0.009 (3)	*
50 µM NEM (NEM(1))	-0.097±0.009 (3)	P < 0.01	-0.195±0.012 (3)	*
500 µM NEM (NEM(2))	-0.343±0.022 (3)	P < 0.01		
100 µM tBHP	-0.412±0.016 (3)	P < 0.01	-0.241±0.015 (3)	P < 0.05
5 µM PAO	-0.534±0.011 (3)	P < 0.01	-0.355±0.018 (3)	P < 0.01

The changes in ΔA_{540} were detected within seven minute interval after addition of mitochondria (see Figs. 1d and 1e) and presented as Means ± SEM. The number of experiments showed in parentheses. P-values were accordingly calculated to experiments free of P5P (D) or 100 µM Ca^{2+} (E). Asterisks indicate that statistical difference between appropriate ΔA_{540} values is not statistically significant.
